# Development, validation and reliability of a questionnaire for assessment of physician's knowledge, attitude and practices (KAPs) regarding foodborne diseases in the Kingdom of Saudi Arabia

**DOI:** 10.12688/f1000research.52997.1

**Published:** 2021-06-14

**Authors:** Mohammed AL-Mohaithef

**Affiliations:** 1 Department of Public Health,College of Health Sciences, Saudi Electronic University, Riyadh, post code: 11673, P.O.Box: 93499, Saudi Arabia

**Keywords:** Content validity; foodborne illness; knowledge, attitudes, and practices (KAPs); physician; reliability; Saudi Arabia

## Abstract

**Background:** The burden of foodborne illness is considered to be high across the world.  Based on the Knowledge-Attitude-Practice (KAP) model, physician’s awareness is essential for conducting individualized treatments, thus reducing the burden of foodborne illness. However, there have been no validated questionnaires specific to the awareness of physicians with foodborne diseases. This study aims to develop and validate a KAP questionnaire for physicians to assess their awareness about the diagnosis and management of foodborne illness.

Methods The questionnaire was developed in three phases: a comprehensive literature review, face and content validity, followed by a reliability test by internal consistency. A cross-sectional study was designed in Abha, Saudi Arabia. Physicians (n=125) were opportunistically recruited from both public and private primary healthcare centers. The questionnaire’s content and validity were confirmed by experts in their corresponding fields. After signing the informed consent, the study participants received the questionnaire to evaluate their KAPs on foodborne diseases. **Results: **A total of 160 physicians from both public and private primary health care centers were approached to enrol 125 study participants into the survey (response rate 78.13%). Of the 31 items designed for assessing the KAP of physicians on foodborne illnesses, three items were excluded after Cronbach’s α analysis. In total, 29 items were included in the final set of the questionnaire. Results of different validity and reliability analyses suggest the questionnaire has a high face and content validity as well as good reliability in internal consistency and stability. **Conclusions: **This study introduces a newly developed questionnaire with good reliability and validity values that can assess physician’s awareness of foodborne disease. The awareness questionnaire, as a study instrument, had a favourable acceptance among physicians. It is a sound method for evaluating and measuring levels of foodborne disease-related awareness among physicians in Abha, Saudi Arabia.

## Introduction

Foodborne illness is a major, but preventable, public health problem across the world. ^1^ The pattern of foodborne illness has changed dramatically due to the lifestyle and behavioral changes of the population. The frequency of foodborne illness outbreaks is growing because of newly discovered pathogens. ^2^ The WHO has estimated that, globally, 1 in 10 people fall ill yearly and 420,000 die as a result of food contamination. ^3^ However, the most recent data on the burden of foodborne diseases in the Kingdom of Saudi Arabia have not yet been published.

The major concern in the Kingdom of Saudi Arabia is the hajj season, where millions of pilgrims come from different parts of the world. The government is very particular about the quality of food items in the restaurant. However occasionally there have been reports on foodborne disease outbreak due to Salmonella, Bacillus cereus and Staphylococcus aureus. ^4^

There has been a constant increase in the number of food poisoning cases in the past few years in the Kingdom of Saudi Arabia, especially during the summer and the Hajj season. ^5^ According to the World Health Organization (WHO), populations in the developing countries including the Middle East, are more likely at risk of foodborne illness due to the restricted strategies in the disease surveillance and prevention and control programs. After the African and South-East Asian regions, it is estimated that the highest burden of foodborne diseases was reported in the Middle East and North African (MENA) region. ^6^ Several studies, including literature reviews and primary studies related to foodborne illnesses in the Kingdom of Saudi Arabia have been conducted to assess the burden. ^7^^–^^12^

Most of the countries across the globe have some kind of mechanism to report foodborne illness. In the last ten years, countries such as Canada, England, Japan and the United States generated an annual report on the cases of foodborne diseases. It is apparent that the cases of staphylococcus intoxication have decreased in most of the countries except in Latin America, due to high consumption of unpasteurized milk and cream-filled desserts. However, the cases of salmonella increased all over the world and new agents such as Escherichia coli 0157:H7 were reported in many countries. ^13^^,^^14^

Cases of Vibrio vulnificus septicaemias have been increasingly reported during the last few years in hospitals in these regions, especially in the Gulf of Mexico states. The patients are more likely to die if the treatment is not given at the appropriate time. The death associated with foodborne diseases is multifactorial and it is also based on the toxicity of the agents. ^15^

Hepatitis E is one of the major public health issues in the entire world. It is mainly transmitted through contaminated food. The sporadic cases of hepatitis E are widely reported in many industrialized nations in Europe, Asia and North America. The burden due to hepatitis E can be reduced through implementation of an effective disease surveillance program and health education. ^16^

The public awareness of food safety has been increasing in the last few years. Though the national statistics of several countries showed a steady decrease in foodborne illness; still the threat to the population continues due to new emerging pathogens. In order to tackle the new emerging pathogens, the Food and Agriculture Organization (FAO) and the World Health Organization (WHO) have introduced a risk-based concept such as Acceptable Level of Protection (ALOP) and Food Safety Objectives (FSOs). These two organisations started using a risk-based approach primarily for controlling the public health hazards due to food insecurity. They have adopted and utilized risk analysis as their main framework to ensure food safety. Microbiological risk assessment has been carried out by the government authority as a part of evaluation of foodborne microbiological hazards to public health. The government has started to make a decision on microbiological safety based on this Microbiological risk assessment. ^17^

General physicians play an important role in the detection and treatment of foodborne illness as they are the first point of contact and a trusted primary source of information for the general public. ^16^ Primary health centers are the first point of contact of the healthcare delivery system, where people in all countries visit for accessing the healthcare services. ^17^

Physicians play a significant role in communicating public health messages to the patients, as the information given is trusted by the patient. Therefore, the physicians must provide information regarding foodborne diseases, at least to the vulnerable population. However, some physicians may not value the counselling session provided for the patients about the foodborne diseases due to the lack of knowledge on food safety, lack of time and lack of perceived benefit. ^18^

Even though there are common guidelines for the diagnosis and treatment of foodborne illness, these vary across physicians. The diagnosis and treatment procedures also vary between physicians working in public and private primary healthcare centers. The literature indicates that misunderstandings occur between the healthcare providers and the patients due to the absence of cultural knowledge and awareness, as well as through the lack of understanding and flexibility. ^3^

Furthermore, a one-dimensional biomedical perspective is frequently found among the healthcare service providers rather than a holistic perspective for providing patient care. ^5^^,^^7^^,^^8^ The self-administered questionnaire is a tool for assessing the insight and experiences of individuals who work alongside the healthcare organization. It is also considered to be another way for evaluating the acceptability and awareness about the multiplicity in healthcare institutions. ^9^

Validated and reliable instruments are lacking for the measurement of the knowledge, attitudes, and practices (KAPs) regarding foodborne illness among physicians in the Kingdom of Saudi Arabia, as well as in all the Gulf Cooperation Council (GCC) countries. The aim of this study was to construct a valid and reliable instrument for assessing the KAPs of physicians in the Kingdom of Saudi Arabia toward foodborne illness.

## Methods

This is a cross-sectional study conducted in the selected public and private primary health care centers in the city of Abha located in the south western region of Asir in the Kingdom of Saudi Arabia. By using the Raosoft sample size calculator ^19^; the sample size has been calculated with a 5% margin of error, and confidence interval of 95 %, a response distribution of 50% and a total population size of 180 (n = 180). The final sample size was n = 125.

Multistage cluster sampling was used in order to recruit the physicians from the selected public and private primary health care centers. A formal permission was obtained from the ministry of health for conducting this particular study on primary health care centers. Permission from the private primary health care centers was obtained separately in order to collect the data for this study.

Physicians in the selected public and private primary health care centers in the city of Abha were approached. Individuals who agreed to participate and had given written consent were included in this study.

## Questionnaire development

There were various phases involved in developing the KAP tool for confirming the validity and reliability. The items in the instrument were designed by a group of experts from the fields of nutrition, microbiology and public health during the first phase. The pilot study, in addition to face, content, and construct validity (primarily designed for validating the structure of the questionnaire) was carried out to determine the validity and reliability of the questionnaire. Factor analysis and the test-retest reliability were performed to ensure the internal consistency and the stability of the questionnaire. Same study participants were asked to complete the questionnaire for the second time within the interval of seven days, to measure the test-retest reliability.

## Evaluating item clarity

The questionnaire was distributed to 125 physicians from both the public and private healthcare sectors to collect information about foodborne illness. After collecting the completed questionnaires, each response was examined carefully looking at the consistency of the answers indicating the questions were widely comprehensible. The questionnaire was then modified to enhance its clarity.

## Evaluating face validity

To appraise the face validity of the questionnaire, ten experts from the Department of Public Health, College of Health Sciences, Saudi Electronic University were selected based on their research experience. They were provided with the questionnaire and the specific objectives of this research. The experts were asked to provide their opinions about the questionnaire related to these research objectives. The feedback from the experts regarding the questionnaire was accepted and incorporated to endorse the face validity.

## Evaluating content validity

To improve content validity, the experts were randomly selected from the authors listed in the publication derived from the Pubmed using key word “foodborne illnesses” and were invited to evaluate the content of the questionnaire. The specialist team included public health professionals, microbiologists, and nutritionists. Their recommendations and perspectives were deliberated and integrated into the questionnaire, thereby helping to establish the content validity of the questionnaire.

The final version of the questionnaire contains information about the KAPs related to foodborne illness and it is available as an extended data. ^20^ Knowledge, in this paper, refers to the accurate technical knowledge among the physicians about foodborne illness, whereas attitude represents the perceptions of physicians regarding foodborne illness. Practices denote the existing management and treatment modalities that the physicians follow when handling food poisoning cases. The questionnaire comprises of four sections. Section one consists of eight questions on collecting various socio-demographic information from the physicians; section two consists of seven questions for determining the knowledge level among the physicians about foodborne diseases; section three consists of seven questions describing the perceptions of physicians about foodborne illness; and section four consists of ten questions for evaluating the current practices of physicians related to the diagnosis and management of foodborne diseases.

## Data analysis

The data analysis was carried out using SPSS (Version 16.0, SPSS Inc. Chicago, IL, USA). The raw data of the food Poisioning study has been deposited as underlying data (v5.5). ^21^ The internal consistency of the questionnaires was determined using Cronbach's α at a satisfactory level that varied between 0.70 and 0.95. ^22^^,^^23^ Pearson's correlation coefficient was used to calculate the test-retest reliability. Cross tabulation and the kappa measure of agreement (k) were used to measure the test-retest agreement of the KAP questionnaire. A two-sided p-value <0.05 was considered statistically significant. As per Cade et al. large correlation coefficients were defined as 0.5 or greater, which indicated higher reliability. ^24^^,^^25^ The strength of agreement was calculated using kappa, and according to Masson et al., the kappa measure of agreement (k) can be classified as follows: poor (<0.20), fair (0.21–0.40), moderate (0.41–0.60), good (0.61–0.80), and very good (0.81–1.00). ^26^

## Ethical considerations

Ethical permission was obtained from the research ethics committee from the College of Medicine, King Khalid University, Abha, Kingdom of Saudi Arabia (REC#2017-03-08). The study was conducted in Abha, Asir Region, where King Khalid University is a designated institutional ethical committee for providing approval for all the research that are confined to the Asir Region. Written consent was obtained from all study participants. Anonymized data were used for analysis and interpretation. The questionnaire and the consent form were securely stored in locked file cabinets and access was given to the specific researchers involved in data analysis.

## Results

A total of 125 from 160 physicians who participated in this study were selected from the government and private primary healthcare centres in the Abha city, with a response rate of 78.13%. Table 1 shows the demographic information of the study participants.

**Table 1.** Demographic characteristic of study participants (n = 125).

Characteristics	n (%)
**Age**	
25-34	18 (14.4)
35-44	52 (41.6)
45-54	42 (33.6)
>54	13 (10.4)
**Gender**	
Male	86 (68.8)
Female	39 (31.2)
**Area of speciality**	
Emergency medicine	20 (16.0)
Family medicine	44 (35.2)
Internal medicine	17 (13.6)
Paediatric medicine	23 (18.4)
OB/GYN	6 (4.80)
Others	15 (12.0)
**Primary setting of practice**	
Government hospital	62 (49.6)
Private hospital	63 (50.4)
**Experience (years)**	
<5 years	29 (23.2)
6-10 years	38 (30.4)
11-15 years	30 (24.0)
>15 years	28 (22.4)
**Number of patients visited per week**	
1-10	6 (4.8)
11-25	17 (13.6)
26-50	21 (16.8)
51-75	13 (10.4)
>75	68 (54.4)
**Number of patients visited with a foodborne illness**	
1-5	68 (54.4)
6-20	40 (32.0)
21-50	13 (10.4)
51-100	1 (0.8)
>100	3 (2.4)

Majority of the physicians (41.6%) were in age group of 35-44 years, 68.8% were male and 35.2% practice family medicine. Majority of physicians (54.4%) reported practising experience of 6-15 years.

Table 2 provides scale statistics for the 31 items. The mean value was 65.33 with a variance of 95.18 and a standard deviation of 9.39. The reliability values were estimated using Cronbach's α. The Cronbach's α, which represents the raw or unstandardized value of alpha, was 0.723. However, Cronbach's alpha, which is based on standardized items, was 0.704; thus, the inference showed that the stronger items are inter-related. From the 31 items that were included in the analysis for reliability, three questions were not in the acceptable range of Cronbach's alpha. These were excluded in the main study. These results prove that the tests are expected to be reliable.

**Table 2.** Internal consistency of knowledge, attitude and practice domain items (n = 125).

Q.No	Scale mean if item deleted	Scale variance if item deleted	Corrected item-total correlation	Cronbach's alpha if item deleted
**Socio-demographic domain**				
1	67.87	103.267	.040	.744
2	68.67	105.667	−.147	.748
3	67.47	101.552	.026	.755
4	67.67	99.381	.172	.740
5	65.73	103.781	−.038	.756
6	68.00	97.571	.213	.739
7	69.40	108.257	−.387	.755
**Knowledge domain**				
8	69.27	106.495	−.241	.750
9	69.67	104.524	−.033	.745
10	69.80	100.314	.475	.732
11	69.73	103.495	.078	.687
12	69.53	106.695	−.237	.751
13	69.27	109.067	−.507	.756
14	68.87	102.267	.286	.737
15	67.87	98.981	.227	.736
**Practice domain**				
16	68.13	104.981	−.077	.746
17	68.33	92.238	.600	.654
18	67.93	94.638	.474	.723
19	68.33	89.952	.731	.707
20	67.87	92.410	.635	.714
21	67.67	92.381	.725	.712
22	67.20	102.886	.163	.739
23	67.27	101.210	.240	.737
24	68.13	90.124	.709	.708
25	68.20	91.029	.697	.710
**Attitude domain**				
26	67.80	92.743	.596	.716
27	67.47	109.838	−.294	.767
28	67.20	115.314	−.495	.781
29	67.80	95.600	.543	.643
30	68.60	103.686	.047	.743
31	68.40	100.829	.093	.746
32	68.07	90.781	.522	.716

Table 3 shows the results from the test-retest analysis using the Pearson correlation coefficient (r). Correlation coefficients were very high for the majority of questions (> 0.7 for 29/31 questions) in the questionnaire. The highest correlation was observed for gender and the lowest correlation was observed for the target microorganisms in canned goods. According to the results from the bivariate correlations, the highest and lowest agreements from test to retest were observed for the same variables as for the Pearson correlation coefficient. Twenty questions from the questionnaire showed a very good strength of agreement, and the remaining nine questions had a good strength of agreement.

**Table 3.** Test-retest reliability of knowledge attitude and practice domain items (n = 125).

Question number	Pearson correlation coefficient (r)	Kappa measure of agreement
**Socio-demographic domain**		
1	0.91	0.82
2	1.0	1.0
3	0.92	0.82
4	0.87	0.78
5	0.93	0.86
6	0.91	0.82
7	0.83	0.74
**Knowledge domain**		
8	0.88	0.80
9	0.89	0.80
10	0.90	0.79
11	0.68	0.51
12	0.82	0.73
13	0.77	0.69
14	0.93	0.86
15	0.91	0.82
**Practice domain**		
16	0.90	0.81
17	0.69	0.60
18	0.90	0.81
19	0.88	0.80
20	0.83	0.74
21	0.79	0.70
22	0.85	0.76
23	0.90	0.81
24	0.93	0.86
25	0.91	0.82
**Attitude domain**		
26	0.87	0.78
27	0.92	0.82
28	0.88	0.80
29	0.67	0.58
30	0.92	0.82
31	0.90	0.81
32	0.87	0.76

## Discussion

A study was conducted among 125 physicians from selected governmental and non-governmental primary healthcare centres in Abha city to evaluate the KAPs among physicians toward foodborne illness. The content and face validity assessments were completed by specialists in related fields, inclusive of microbiologists and public health practitioners. Item clarity was tested by conducting a pilot study among these 125 physicians. The same evaluations of the face and content validity were performed in a study conducted in Malaysia to validate the questionnaire on KAPs related to lifestyle. ^27^ The internal reliability between the questions was calculated by using Cronbach's α. Our KAP questionnaire on foodborne illness showed adequate internal uniformity (α = 0.723). Numerous previously published studies on Cronbach's α have projected a satisfactory α value that varied between 0.70 to 0.95 for this test. ^28^^,^^29^ The results of the present study, α = 0.723, are supported by the values mentioned in recently published research articles. ^30^ A study conducted in Iran for assessing the KAPs of disaster preparedness reported an internal consistency of α = 0.785, which is somewhat greater than the current study. ^30^ Another study conducted in Malaysia for assessing the KAPs related to dengue fever prevention, also demonstrated internal consistency with Cronbach's α = 0.798, which is higher than the results of the present study. ^22^A similar value of Cronbach's α was obtained in a study aimed at measuring the KAPs for the prevention of dengue fever among the male population in the Maldives. ^23^ A study among Iraqi patients about the KAPs related to immunization, reported an internal consistency with Cronbach's α score of 0.812, which is also higher than that observed in our study. ^31^

A study conducted in Spain for assessing the development and validation of the questionnaire related to academic stress in secondary education found that the internal consistency Cronbach's α value is 0.77, which is slightly higher than the current study. ^32^ Similarly, a study in Malaysia of a KAP questionnaire among the childcare providers reported a Cronbach's α coefficient ranging between 0.89 to 0.90, which is much higher than the present study. ^33^

Likewise, a study conducted in Iran for evaluating the KAPs related to obesity among adults and adolescents revealed a Cronbach's α score of 0.72, which is similar to the current study. ^34^ A similar study conducted among Iranian adults regarding the KAPs related to oral health reported a Cronbach's α of 0.82, which is higher than the value reported in this study. ^35^ To determine the KAPs regarding vitamin D among adults in Tehran, Amiri P. et al. presented an internal consistency value of 0.60, which is less than recorded in our study. ^36^

The current study results showed that the correlation coefficient was high for many questions in the KAPs questionnaire about foodborne illness. The results of a study by Rachel et al. ^37^ on the test-retest reliability of a new questionnaire on diet and eating behaviors, found that the correlation coefficient was high for most questions, which supports the results of this study.

The present study indicated that 29 of 32 items had a good level of reliability. Similarly, a study conducted by Luísa. et al. ^38^ in Brazil on the reliability of a questionnaire for the assessment of food safety knowledge, perceptions, and practices found significance in stability and reliability of the questions. Comparable results were observed in the studies that were conducted previously about the knowledge, perceptions and behaviors related to food safety, which also support the results of the current study. ^39^^,^^40^

There are certain limitations in this study. Although the study had representative samples of physicians from both public and private primary health care centers; the sample was drawn only from one particular region (Asir region) in the Kingdom of Saudi Arabia. Self-reported questionnaire was the primary source for collecting the information from the physicians, therefore, the quality of information delivered by the study participants is arguable.

## Conclusion

A questionnaire was framed and developed to evaluate the KAPs among physicians toward foodborne illness. It was shown to have adequate validity and reliability. This questionnaire can be classed as a noteworthy instrument for assessing the KAPs related to foodborne illness among physicians across the country. The study highlights the importance of a standardized questionnaire to assess KAPs among physician towards foodborne illness to initiate an appropriate interventional program for physicians to reduce the burden of foodborne illnesses.

## Data availability

### Underlying data

Harvard Dataverse, V1: Data-Food Poisoning. http://doi.org/10.7910/DVN/QP1KVI21. ^21^

The project contains the following underlying data:

[Data-Food Poisioning] (Raw, unaveraged qPCR data).

### Extended data

Harvard Dataverse, V1: “Data-Food Poisioning”.

https://doi.org/10.7910/DVN/G67UW3. ^20^

This project contains the following extended data:

• Annexure 1: Questionnaire on Socio-demographic Variables
• Annexure 2: Questions regarding the knowledge, attitude and practices about food borne illness

Data are available under the terms of the Creative Commons Zero “No rights reserved” data waiver (CC0 1.0 Public domain dedication).
