# Supplementary material for: Development, validation and reliability of a questionnaire for assessment of physician's knowledge, attitude and practices (KAPs) regarding foodborne diseases in the Kingdom of Saudi Arabia
Source: F1000Res. 2021 Sep 20;10:470. Originally published 2021 Jun 14. [Version 2] doi: 10.12688/f1000research.52997.2 (PMC8630547; doi:10.12688/f1000research.52997.2)
Supplement: Peer review history

# Peer review: Reviewer response for version 2

**DOI:** 10.5256/f1000research.77125.r100544

**Published:** 2021-11-29

**Authors:** Khaled Hamden

**Affiliations:** 1 Laboratory of Bioresources: Integrative Biology and Valorization, Higher Institute of Biotechnology of Monastir, University of Monastir, Monastir, Tunisia

This  paper submitted to your journal evaluated to possible methods of reducing the burden of foodborne illness. In this study, the author to try to develop and validate a KAP questionnaire in order to assess, diagnose and take charge of foodborne illness. This study was carried out on 125 study participants.

 This manuscript is well structured and which deals with a topical subject and which deals with a real problem which affects the people to the whole world. I give a favorable opinion for the indexing of this manuscript without changes. After reading, my conclusion is that this version has been revised well and the responses to the comments and suggestions of the reviewers are clear and therefore this version can be accepted for indexing in this form.

Is the work clearly and accurately presented and does it cite the current literature?

Yes

If applicable, is the statistical analysis and its interpretation appropriate?

Yes

Are all the source data underlying the results available to ensure full reproducibility?

No source data required

Is the study design appropriate and is the work technically sound?

Yes

Are the conclusions drawn adequately supported by the results?

Yes

Are sufficient details of methods and analysis provided to allow replication by others?

Yes

Reviewer Expertise:

Food and human diseases

I confirm that I have read this submission and believe that I have an appropriate level of expertise to confirm that it is of an acceptable scientific standard.

# Peer review: Reviewer response for version 1

**DOI:** 10.5256/f1000research.56337.r87510

**Published:** 2021-06-29

**Authors:** Nazik Eltayeb Musa Mustafa

**Affiliations:** 1 Department Food Hygiene and Safety, Faculty of Public and Environmental Health, University of Khartoum, Khartoum, Sudan

**General comments:**

- This is a significant study concerned with the development of tools used to investigate knowledge, attitude, and practices of physicians engaged in the diagnosis and treatment of food-borne diseases.
- The study tackles important issues related to food safety KAP and it participates actively in providing knowledge about physician food safety KAP. However, food safety descriptive findings related to knowledge attitude and practices could be included in the manuscript to maximize the benefit of this research for instance the descriptive data could be extended beyond the demographic data to Include (concisely) the major findings of physician knowledge attitude and practices instead of presenting them only in the forms of Internal consistency and test-retest reliability.
- Nevertheless, the study gives a deep inside look at the construction of the questionnaire in terms of reliability and validity and it gives substantial weight to general questionnaire construction techniques.

**Specific comments:**

***Abstract:***

- The phrase ‘Physicians (n=125) were opportunistically recruited’ does not clearly reflect the multistage cluster sampling technique applied for selected public and private primary health care centers mentioned in the method section

***Introduction:***

1. In the 2nd paragraph, line 2 the sentence ‘The government is very particular about the quality of food items in the restaurant; may need modification to reflect more generalized food control activities i.e., food establishments instead of the phrase ‘the restaurant’.
2. In the same paragraph bacterial names *Bacillus cereus* and *Staphylococcus aureus* should be in italics (there is no need to italicize the name Salmonella).
3. The bacterial names *Escherichia coli* (paragraph 4 line 5) should also be italicized.
4. Same should be done for *Vibrio vulnificus* (paragraph 5 line 1).
5. Paragraph 6 needs revision to specify the Saudi government, a slight reordering of the sentences in this paragraph will increase its readability.
6. I suggest merging Paragraphs 7 and 8 to avoid redundancy in specifying the importance of the Physicians roles in food safety.

***Methods:***

- A slight change in the abstract is needed to reflect the methods followed in selecting samples.
- It appears to the reader that evaluating face validity and evaluating content validity for the questionnaire were done in the same way, hence no need to repeat the sentences, I suggest merging the evaluation of face and validity.
- In the sentence ‘experts were randomly selected from the authors listed in the publication derived from the PubMed using the keyword “foodborne illnesses”'. It is not clear if the author includes Saudi Arabia among search keywords? More elaboration is needed here about the responders’ number and distribution in fields.

***The questionnaire:***

 In annexure 2:

- Some of the questions, for example, question 13 could have had been asked with less ambiguity for example question 13 (The target microorganism in canning is?) could be asked in a more straightforward way (i.e. The most encountered food poisoning microorganisms from canned food is?).
- Some of the questions regarding the knowledge, attitude, and practices about foodborne illness are barely related to food safety issues generally tackled by physicians, for example, question 19 is quite lengthy and hardly fulfills the required target.

***Results and discussion:***

- These are professionally written sections with clear tables and main finding paragraphs.
- I could not find the descriptive results of the findings related to knowledge, attitude, and practices of physicians towards food safety. It seems that it is the author's preference not to present descriptive data in this regard. Adding such data will benefit readers from the field with fewer statistics knowledge and increase the manuscript audience.

Is the work clearly and accurately presented and does it cite the current literature?

Yes

If applicable, is the statistical analysis and its interpretation appropriate?

I cannot comment. A qualified statistician is required.

Are all the source data underlying the results available to ensure full reproducibility?

Yes

Is the study design appropriate and is the work technically sound?

Yes

Are the conclusions drawn adequately supported by the results?

Partly

Are sufficient details of methods and analysis provided to allow replication by others?

Yes

Reviewer Expertise:

Food safety, bacteriology, Public health

I confirm that I have read this submission and believe that I have an appropriate level of expertise to confirm that it is of an acceptable scientific standard.

# Response

**Published:** 2021-08-26

**Authors:** Mohammed AL-Mohaithef

**Affiliations:** Saudi Electronic University , Saudi Arabia

We have incorporated the reviewer comments and uploaded the new version of the manuscript.
